# Bimetallic Perthiocarbonate Complexes of Cobalt: Synthesis, Structure and Bonding

**DOI:** 10.3390/molecules29112688

**Published:** 2024-06-06

**Authors:** Alaka Nanda Pradhan, Shivankan Mishra, Urminder Kaur, Bikram Keshari Rout, Jean-François Halet, Sundargopal Ghosh

**Affiliations:** 1 Department of Chemistry, Indian Institute of Technology Madras, Chennai 600036, India; salupradhan7@gmail.com (A.N.P.); shivankan1994@gmail.com (S.M.); urminderkaur27@gmail.com (U.K.); bikramrout44@gmail.com (B.K.R.); 2 Univ Rennes, CNRS, École Nationale Supérieure de Chimie de Rennes, Institut des Sciences Chimiques de Rennes (ISCR)—UMR 6226, F-35000 Rennes, France

**Keywords:** chalcogen, cobalt, perthiocarbonate, sulfur, trithiocarbonate, vanadium

## Abstract

The syntheses and structural elucidation of bimetallic thiolate complexes of early and late transition metals are described. Thermolysis of the bimetallic hydridoborate species [{Cp*CoPh}{*µ*-TePh}{*µ*-TeBH_3_-*ĸ*^2^Te,H}{Cp*Co}] (Cp* = *ɳ*^5^-C_5_Me_5_) (**1**) in the presence of CS_2_ afforded the bimetallic perthiocarbonate complex [(Cp*Co)_2_(*μ*-CS_4_-*κ*^1^S:*κ*^2^S′)(*μ*-S_2_-*κ*^2^S″:*κ*^1^S‴)] (**2**) and the dithiolene complex [(Cp*Co)(*μ*-C_3_S_5_-*κ*^1^S,S′] (**3**). Complex **2** contains a four-membered metallaheterocycle (Co_2_S_2_) comprising a perthiocarbonate [CS_4_]^2−^ unit and a disulfide [S_2_]^2−^ unit, attached opposite to each other. Complex **2** was characterized by employing different multinuclear NMR, infrared spectroscopy, mass spectrometry, and single-crystal X-ray diffraction studies. Preliminary studies show that [Cp*VCl_2_]_3_ (**4**) with an intermediate generated from CS_2_ and [LiBH_4_·THF] yielded thiolate species, albeit different from the cobalt system. Furthermore, a computational analysis was performed to provide insight into the bonding of this bimetallic perthiocarbonate complex.

## 1. Introduction

The efficiency of metal sulfides as heterogeneous catalysts and the activity of metallo-proteins in enzyme-mediated catalysis are generally significantly impacted by the nature of the metal–sulfur entities. An intriguing and unusual structural chemistry became apparent via a systematic search for metal–sulfur types of compounds given the variety of coordination possibilities that sulfur ligands can provide [1,2]. Indeed, CS_2_ ligands have been shown to be versatile synthetic reagents ever since the first transition metal–CS_2_ complexes were synthesized in 1967 [3] due to their electron donating and electron accepting properties, yielding an array of binding modes with one or more transition metal atoms [4,5,6].

**Chart 1:**
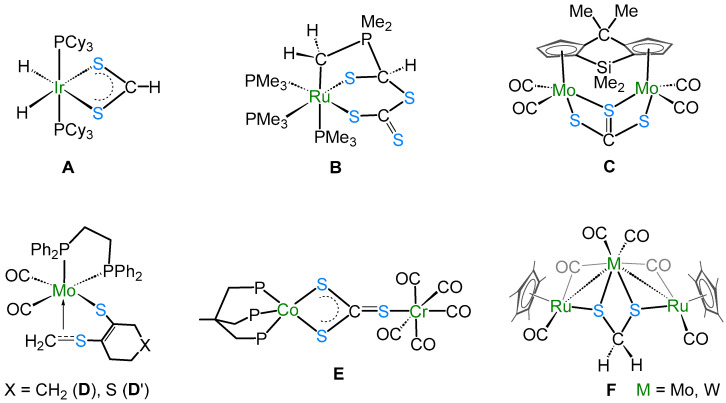
Different binding modes of CS_2_-based ligands (**A**–**F**) in transition metal complexes.

In this context, Jagirdar’s group demonstrated the insertion of CS_2_ into one of the Ir-H bonds in [Ir(H)_5_(PCy_3_)_2_] to afford the dihydrido dithioformate complex *cis*-[Ir(H)_2_(*ƞ*^2^-S_2_CH)(PCy_3_)_2_] (**A**), accompanied by the elimination of H_2_ [7]. Similarly, the cyclometalated ruthenium complex [Ru(S_2_C(H)PMe_2_CH_2_-*κ*^3^S,S,C)(PMe_3_)_3_] was shown to react with an excess of CS_2_, which undergoes a second insertion into a Ru–S bond to yield [Ru(SC(=S)SCH(–S)PMe_2_CH_2_-*κ*^3^S,S,C)(PMe_3_)_3_] (**B**) [8]. Wang and coworkers reported the disproportionation of CS_2_ when reacted with the Me_2_C and Me_2_Si doubly bridged bis(cyclopentadienyl) dinuclear molybdenum carbonyl complex (Me_2_C)(Me_2_Si)[(*ƞ*^5^-C_5_H_3_)Mo(CO)_3_]_2_, which led to the formation of complex **C** [9]. Likewise, in 2021, Fischer’s group showcased that the nucleophilicity of the monodithiolene species [Mo(CO)_2_(dt)(dppe)] (dt: cydt = cyclohex-1-ene-1,2-dithiol or tpydt = 5,6-dihydro-2H-thiopyran-3,4-dithiol, dppe = 1,2-bis(diphenylphosphino)ethane) can be switched on by reduction with 2 eq. of [Co(Cp*)_2_] in a mixture of DCM and ACN, which leads to the isomorphic complexes [Mo(CO)_2_(CH_2_-dt)(dppe)] (dt = cydt (**D**); dt = tpydt (**D′**)), with molybdenum formally in the zero oxidation state [10]. In addition, Bianchini’s group showcased the nucleophilic nature of *ƞ*^2^-CS_3_, where the trithiocarbonate complex of cobalt [Co(tppme)(S_2_CS)] (tppme = 1.1.1-tris(diphenylphosphinomethyl)ethane) was reacted with [Cr(CO)_5_·THF], resulting in the formation of **E** [11]. Recently, our group has reported the reaction of *arachno*-ruthenaborane [(Cp*Ru)_2_(B_3_H_8_)(CS_2_H)] with group 6 metal carbonyls [M(CO)_5_·THF] (M = Mo and W), which led to the formation of methanedithiolato-bridged hetero-trimetallic complexes, namely [{Cp*Ru(CO)}_2_(*μ*-CO)_2_(*μ*_3_-CH_2_S_2_-*κ*^2^S′:*κ*^2^S′′){M(CO)_2_}] (M = Mo and W) (**F**) [12] (Chart 1).

Over the last few decades, we and others have synthesized interesting polyhedral clusters, both single-cage and fused, employing early and late transition metals [13,14,15,16,17,18]. Despite being largely uncontrolled, the isolation of numerous unique metallaheteroboranes demonstrating novel coordination modes prompted us to investigate this area more meticulously. In this context, our group utilized three distinct strategies: (i) pentamethylcyclopentadienyl-based metal halides with chalcogen-based borate ligands such as [LiBH_2_E_3_], [LiBH_3_EPh] (E = S, Se, Te), [LiBH_4_ + CS_2_], or [NaBH_4_ + CS_2_] [19,20,21]; (ii) preformed metallaboranes with the above chalcogen-based borate ligands [21]; and (iii) preformed metallaboranes with CS_2_ or CS_2_-based ligands such as [CS_2_·PPh_3_] [22,23]. In our earlier reports, the classical diborane [B_2_H_5_]^−^ was stabilized by the reaction of [Cp*TaCl_4_] with [LiBH_4_·THF], followed by the addition of excess [S_2_C·PPh_3_] adduct [23]. Also, these chalcogen-based borate ligands have been utilized for cluster expansion reactions. The reactivity of *nido*-[(Cp*M)_2_B_6_H_10_] (M = Co and Rh) with the chalcogen-based borate Li[BH_2_E_3_] (E = S, Se, or Te) afforded 10-vertex *nido*-dimetallaheteroborane clusters [21]. By using a different chalcogen-based ligand (intermediate generated from CS_2_ and [LiBH_4_·THF]) with *nido*-[(Cp*M)_2_B_6_H_10_] (M = Co and Rh), unique dimetalladecaborane(14) analogues containing di(thioboralane)-thione {B_2_CS_3_} and di(thioborolane) {B_2_S_2_CH_2_} moieties were isolated [21]. Very recently, the reactivity of a borate ligand derived from CS_2_ with *κ*^2^-N,S-chelated ruthenium borate complexes, [Ph_3_P(*κ*^2^-N,S-L)Ru{*κ*^3^-H,S,S′-H_2_B(L)_2_}] (L = C_7_H_4_NS_2_, C_5_H_4_NS), has been investigated [24]. Therefore, we pursued this chemistry, reacting the cobalt borate complex [{Cp*CoPh}{*µ*-TePh}{*µ*-TeBH_3_-*ĸ*^2^Te,H}{Cp*Co}] (**1**) [19] with CS_2_. Although the objective of obtaining transition metal complexes containing boron content was not achieved, interesting cobalt and vanadium thiolate complexes were isolated.

## 2. Results and Discussion

### 2.1. Reactivity of ***1*** with CS_2_

**Scheme 1:**
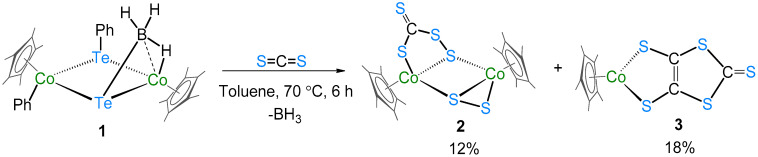
Synthesis of the perthiocarbonate (**2**) and dithiolene (**3**) complexes of cobalt.

Transition metal–chalcogen complexes are well documented in the literature. Recently, we have shown that the reaction of [Cp*Ru(*μ*-Cl)Cl]_2_ with Na[BH_3_(SCHS)] led to the formation of several bimetallic dithioformato ruthenium complexes, where the dithioformate ligand (CH_2_S_2_) shows diverse binding modes with the metal center(s) [20]. In addition, the reaction of [1,2-(Cp*Ru)_2_(*μ*-H)_2_B_3_H_7_] with CS_2_ yielded the *arachno*-ruthenaborane [(Cp*Ru)_2_(B_3_H_8_)(CS_2_H)], with a dithioformato ligand attached to it by the metal-assisted hydroboration [22]. Thus, investigation of the CS_2_ ligand with transition metal boron species became of interest. With this background, we reacted **1** with CS_2_ under thermolytic condition. Thus the reaction of **1** with CS_2_ under thermolytic conditions. The reaction of **1** with CS_2_ in toluene at 70 °C for 6 h led to the formation of an air-stable complex, the brown solid **2** (18% yield, R*_f_* = 0.46), and the known green dithiolene complex [(Cp*Co)(*μ*-C_3_S_5_-*κ*^1^S:*κ*^1^S′] **3** [25] (12% yield, R*_f_* = 0.58) (Scheme 1). The identification of complex **2** was confirmed by combined ^1^H, ^13^C{^1^H} NMR, IR spectroscopy, mass spectrometry, and single-crystal X-ray diffraction studies. The detailed characterization of complex **2** is discussed below.

The ^11^B NMR of complex **2** shows no chemical shifts, and the ^1^H NMR spectrum shows no upfield region peaks, which rules out the possibility of the formation of a boron-containing complex. Indeed, complex **2** is formed by the release of BH_3_ from complex **1**. We confirmed the release of the [BH_3_·PPh_3_] adduct by trapping it with PPh_3_. The ^11^B{^1^H} and ^31^P{^1^H} NMR spectra (Figures S7 and S8) of the reaction mixture show resonances at *δ* = −39.3 and 20.8 ppm, respectively. The ^1^H NMR spectrum displays two resonances at *δ* = 1.38 and 1.63 ppm at a 1:1 ratio, corresponding to Cp* ligands. The presence of two different Cp* ligands is also supported by the ^13^C{^1^H} NMR spectrum. In addition to Cp* peaks, the ^13^C{^1^H} NMR shows resonance at *δ* = 252.7 ppm, which indicates the presence of a C=S moiety. The infrared spectrum shows a peak at 1020 cm^−1^ that confirms the presence of this C=S double bond. Further, the mass spectrometric analysis of **2** shows an isotopic pattern (ESI^+^ mode) at *m*/*z* = 592.9376, which corresponds to the molecular formula C_21_H_31_Co_2_S_6_. Although all the spectroscopic and mass spectrometric data point toward the formation of a thiolate complex, the solid-state framework of **2** was confirmed by single-crystal X-ray diffraction studies. The crystals were grown by slow evaporation of an *n*-hexane/CH_2_Cl_2_ (30:70) solution at 5 °C.

**Figure 1:**
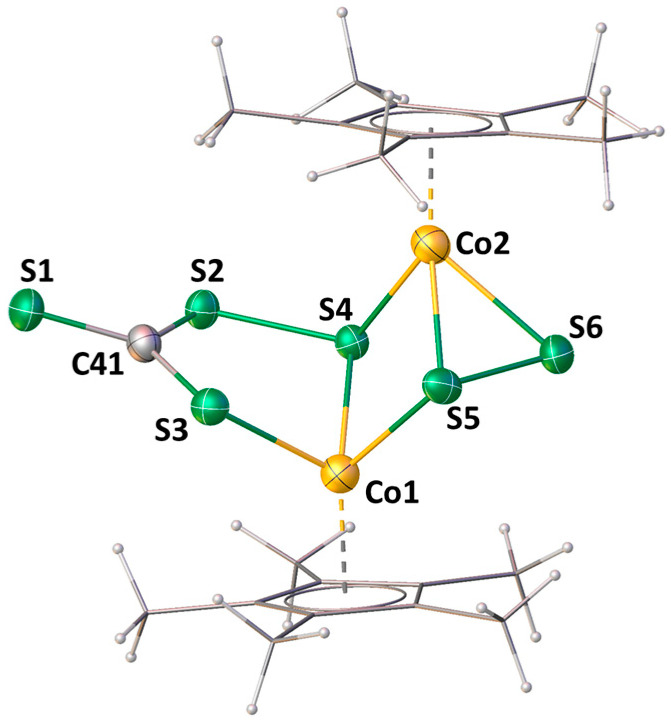
Molecular structure and labeling diagram of **2**. Selected bond lengths (Å) and bond angles (°): Co1–S4 2.200(3), Co1–S3 2.264(4), S5–S6 2.043(14), Co2–S5 2.202(4), Co2–S6 2.258(15), Co1···Co2 3.332, C41–S1 1.663(10), C41–S2 1.724(12), C41–S3 1.722(10), S2–S4 2.080(4), S3–C41–S2 119.3(6), Co1–S4–Co2 95.97(12), S4–Co1–S5 83.08(13), S5–Co2–S6 54.5(4), Co2–S5–Co1 96.91(13), S5–S6–Co2 61.4(4).

The solid-state X-ray structure of complex **2** shows the bimetallic perthiocarbonate complex [(Cp*Co)_2_(*μ*-CS_4_-*κ*^1^S:*κ*^2^S′)(*μ*-S_2_-*κ*^2^S″:*κ*^1^S‴)] (Figure 1). Note that there are two molecules in the asymmetric unit. To the best of our knowledge, complex **2** is the first structurally characterized perthiocarbonate complex of cobalt. It consists of a four-membered metallaheterocycle (Co_2_S_2_) comprising a rare perthiocarbonate [CS_4_]^2−^ unit and a disulfide [S_2_]^2−^ unit, attached opposite to each other. The bidentate [CS_4_]^2−^ fragment bridges two {CoCp*} moieties in a *µ*_2_-*ɳ*^2^ fashion. Complex **2** represents, in turn, a novel type of structure having both perthiocarbonate anion [CS_4_]^2−^ and disulfide [S_2_]^2−^ units, with 18-electron cobalt (III) centers. Note that Adams’ group previously showed that the disulfur unit is a potential candidate for disulfur/dithiolate conversion [26,27].

The average Co–S bond length of 2.242 Å is comparable to that in cobalt–thiolate complexes [28,29], and the average bridged Co–S distance of 2.233 Å is slightly shorter than the unbridged Co–S bonds (2.261 Å). Further, the Co∙∙∙Co distance of 3.332 Å clearly suggests the absence of any metal–metal (Co–Co) bond, making it a coordinatively saturated 18-electron species. Transition metal–perthiocarbonate complexes are well documented in the literature, in which the [CE_4_]^2−^ ion (E = S, Se) acts as a 1,1′-dithiolate ligand. Some examples of such complexes are listed in Table 1 [30,31,32,33], which describe the different bonding modes, structural parameters, and spectroscopic data of complexes with [CE_4_]^2−^ units.

Generally, the perthiocarbonate anion, [CS_4_]^2−^, is obtained by the reaction of CS_2_ with disulfide or polysulfide dianions. Perthiocarbonate complexes can be synthesized from trithiolate complexes [M(CS_3_)_2_]^2−^ by I_2_ oxidation or the addition of sulfur [34]. Shieh and co-workers reported the first example of [CE_4_]^2−^ incorporated into transition metal–carbonyl complexes, [(CE_4_)_2_Mn_2_(CO)_6_]^2−^ (E = S, Se) (Table 1) [32], where two [CE_4_]^2−^ fragments were connected to two {Mn(CO)_3_} units symmetrically. In the case of complex **2**, only one [CS_4_]^2−^ unit is present. In this [CS_4_]^2−^ unit, the bond distance C41–S1 (1.663(10) Å) is a bit shorter than C41–S3 (1.722(10) Å) and C41–S2 (1.724(12) Å) and indicates a double-bond character. In **3**, the double-bond character of C=S (1.657(7) Å) is slightly more pronounced than in **2** (1.663(10) Å). On the other hand, the four-electron donor disulfide ligand is coordinated to two cobalt centers in *ɳ*^1^,*ɳ*^2^-S_2_ mode, similar to [(Cp*Co)_2_(*ɳ*^1^,*ɳ*^2^-S_2_)_2_] [35], [(Cp*Ru)_2_(*ɳ*^1^,*ɳ*^2^-S_2_)(*ɳ*^1^,*ɳ*^1^,*ɳ*^1^-S_3_)] [36], and [(triphos)_2_Rh_2_(*ɳ*^1^,*ɳ*^2^-Se_2_)_2_][PPh_4_]_2_ (triphos = H_3_CC(CH_2_PPh_2_)_3_) [37]. The S–S distance of 2.043(14) Å in **2** is comparable to the S–S distances measured in [(Cp*Co)_2_(*ɳ*^1^,*ɳ*^2^-S_2_)_2_] (2.062(6) Å) [35] and [(Cp*Ru)_2_(*ɳ*^1^,*ɳ*^2^-S_2_)(*ɳ*^1^,*ɳ*^1^,*ɳ*^1^-S_3_)] (1.907(5) Å) [36].

**Figure 2:**
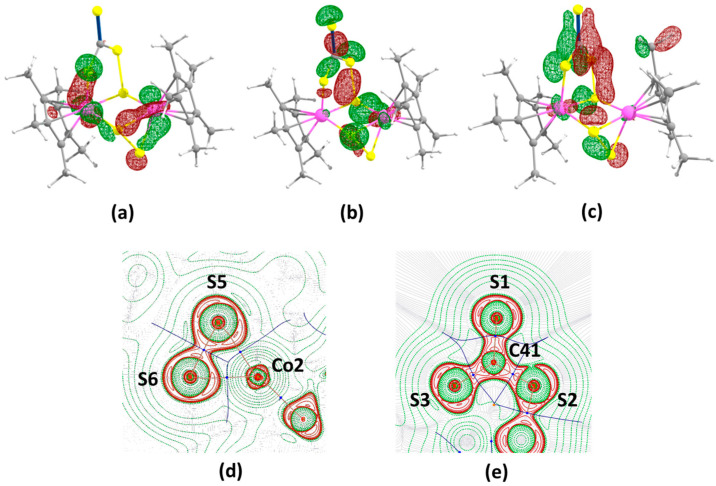
(**a**) HOMO-12, (**b**) HOMO-17, and (**c**) HOMO-26 of **2**. Contour isodensity values for isosurface are ±0.045 (e/bohr^3^)^1/2^. (**d**,**e**), Laplacian maps of the electron density distribution computed for **2** in the Co2–S5–S6 plane and the S1–S2–S3 plane, respectively.

DFT theoretical calculations were carried out for insight into the electronic structure and bonding situation in **2** (see Section 3.3 for the computational details). The molecular orbital (MO) analysis of **2** shows a large HOMO-LUMO energy gap of 3.19 eV. Among the important MOs, the HOMO-12 of **2** shows a large *d* (M)-*p* (S) interaction of atom Co2 with the sulfur atoms of the disulfur unit (Co2–S5 and Co2–S6) (Figure 2a). Moreover, the HOMO-26 of **2** depicts the S–S end-to-end *σ*-bonding interaction of the disulfur unit, which is also confirmed by the contour line diagram (Figure 2c,d). The HOMO-26 also shows a side-to-side overlap of the *p* orbitals of C41–S1 (*π*-interaction). This double-bond character is also reflected by a higher Wiberg bond index (WBI) computed for C41–S1 (1.585 Å) and a shorter calculated bond distance of 1.652 Å. On the other hand, the HOMO-17 of **2** shows the *σ*-bonding interaction of the [CS_4_] unit (Figure 2b). Overall, the contour line diagram reveals the CS_3_ bonding interaction in the S1–C41–S2–S3 plane, which also indicates the *sp*^2^ character of the atom C41 (Figure 2e).

In the recent past, we isolated and structurally characterized paramagnetic thiocarbonate complexes from the reaction of Na_5_[B(CH_2_S_2_)_4_] and [Cp*VCl_2_]_3_ (**4**) [38]. Thus, with the objective of isolating similar types of thiolate complexes to that of complex **2**, we explored the reactivity of [Cp*VCl_2_]_3_ (**4**) with an intermediate generated from CS_2_ and [LiBH_4_·THF] under different reaction conditions, which yielded the inseparable yellow solids **5** and **6** (Scheme S1). Although we do not have detailed characterizations of these inseparable complexes, preliminary studies (X-ray diffraction analysis) show that **5** and **6** are binuclear vanadium trithiocarbonate [(Cp^∗^V)_2_(*μ*-CS_3_-*κ*^2^S,S′)_2_] and (disulfaneyl)methanethiolate [(Cp*V)_2_(*μ*-SCH_2_S_2_-*κ*^2^S,S′)_2_] complexes, respectively (Figures S1 and S2).

### 2.2. UV–vis Study of ***2***

**Figure 3:**
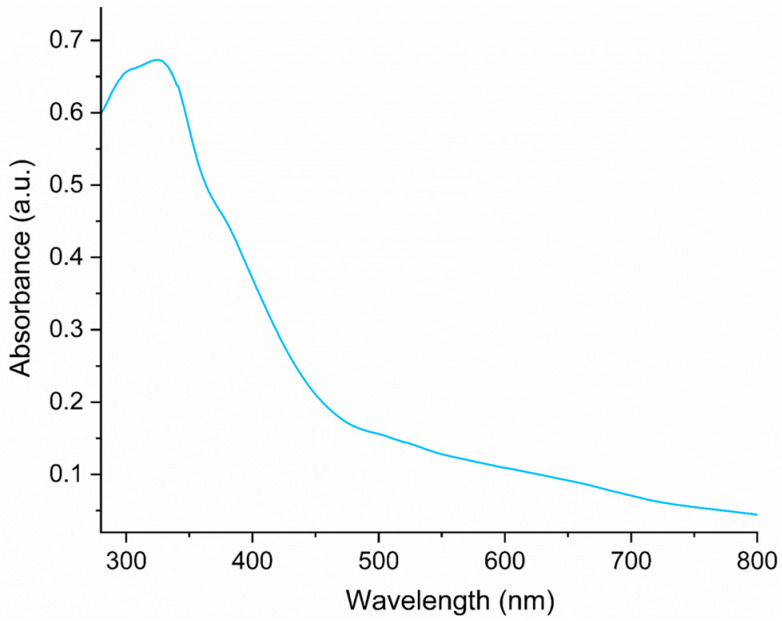
UV–vis spectrum of **2** in CH_2_Cl_2_.

The absorption patterns of the colored complex **2** may be of interest due to the presence of different chalcogen fragments. Therefore, the UV–vis absorption spectrum of **2** was measured in the range of 280–800 nm in CH_2_Cl_2_ solution at 298 K (Figure 3). In fact, the most intense peaks in the higher-energy regions (280–320 nm) are due to the spin-allowed π–π* transition of the Cp* ligands present in these complexes [39]. Comparatively low-intensity peaks at a lower energy around 320–550 nm may be due to the charge transfer bands. In order to obtain more detail, time-dependent DFT calculations were carried out on **2**. In complex **2**, the high-intensity absorption band near 326 nm is due to the HOMO–6→LUMO electronic transition. The HOMO-6 is mostly from metal-based *d* orbitals, while the LUMO has both metal (*d* orbital) and disulfide [S]^2−^ (*p* orbital on S atoms) orbital characters (Figure S10). Therefore, these absorption bands can be assigned as metal-to-ligand charge transfer (MLCT) transitions. In the low-energy wavelength region, **2** shows a low-intensity absorption band at 570 nm, which could be due to the intramolecular LMCT transitions.

### 2.3. Electrochemical Study of ***2***

**Figure 4:**
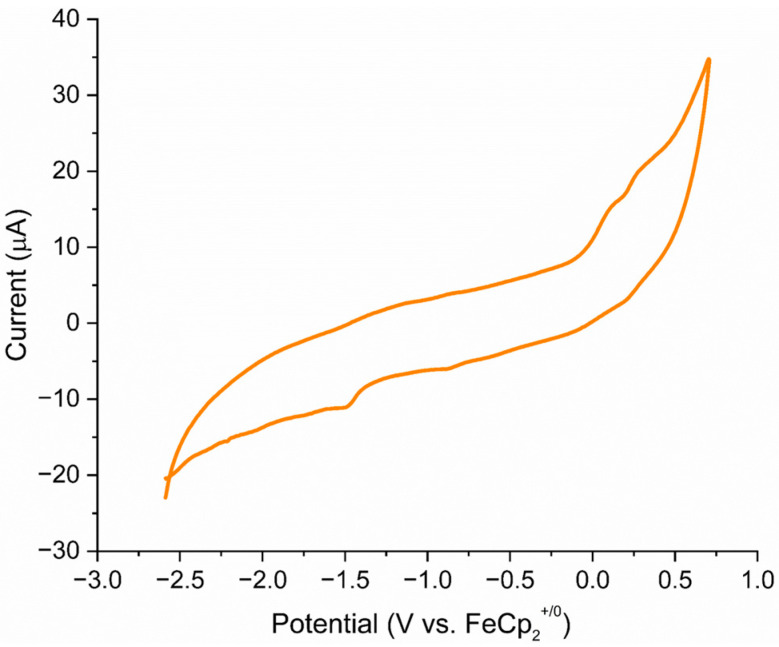
Cyclic voltammogram (CV) recorded for complex **2** in DMF at 298 K.

The redox properties of complex **2** dissolved in DMF were examined using cyclic voltammetry (Figure 4). The DMF solution displays two irreversible responses at *E*_pc_ = −1.47 V and −0.86 V vs. Fc/Fc^+^. They may originate from the reduction of the disulfide or metal-centered reduction based on the reported literature. The two irreversible anodic responses at *E*_pa_ = 0.11 V and 0.27 V vs. Fc/Fc^+^ are most likely due to the oxidation of the thiolate ligand [40,41,42,43,44].

## 3. Materials and Methods

### 3.1. General Procedures and Instrumentation

All the experimental procedures were performed in an argon atmosphere by using standard Schlenk line techniques and a glove box. All the solvents, *n*-hexane, DCM (dichloromethane), toluene, and THF (tetrahydrofuran), were distilled under an argon atmosphere prior to use. All the chemicals, such as Ph_2_Te_2_ (diphenyl ditelluride) (Sigma Aldrich, Bangalore, India) and CS_2_ (Loba Chemie Pvt. Ltd., Mumbai, India), were purchased and used as received. The complexes [{Cp*CoPh}{*µ*-TePh}{*µ*-TeBH_3_-*ĸ*^2^Te,H}{Cp*Co}] (**1**) [19] and [Cp*VCl_2_]_3_ (**4**) [45] were prepared according to the methods from the literature. Dialuminum-supported TLC plates (MERCK TLC plates, Bangalore, India) were used for the separation of the reaction mixtures. All the NMR spectra for the synthesized complexes were obtained on 500 MHz Bruker FT-NMR spectrometers, Billerica, MA, USA. The residual solvent protons (CDCl_3_, *δ* = 7.26 ppm) and carbons (CDCl_3_, *δ* = 77.1 ppm) were employed as references for the ^1^H and ^13^C{^1^H} NMR spectra, respectively. The mass data of all the synthesized complexes were recorded on Q-Tof Micro YA263 HRMS, Milford, MA, USA and 6545 Q-Tof LC/MS instruments, Santa Clara, CA, USA. The IR spectrum of **2** was recorded in dichloromethane solvent with a JASCO FT/ IR-1400 spectrometer, Easton, MD, USA. The UV–vis spectrum was recorded on a JASCO V-650 spectrometer, Tokyo, Japan. The cyclic voltammetry measurement was performed on an OrigaLys potentiostat, Rillieux-la-Pape, France in a standard three-electrode system (glassy carbon working electrode, platinum wire counter electrode, and Ag/Ag^+^ as pseudo reference electrode). For deoxygenation, argon was bubbled into the electrolyte medium for 10 min. The cyclic voltammograms were recorded at a scan rate of 50 mVs^−1^.

**Synthesis of 2**. In a pre-dried Schlenk tube, **1** (0.10 g, 0.123 mmol) was suspended in 10 mL of toluene. Under the atmosphere of argon, CS_2_ (4 mL) was added slowly using a syringe at room temperature to the green solution of complex **1**. Then, the reaction mixture was kept at 70 °C (temperature of oil bath) for 6 h with continuous stirring under an atmosphere of argon. The solvent was removed by vacuum, and the remaining residue was separated and purified using the TLC method on silica gel TLC plates. Elution with *n*-hexane/CH_2_Cl_2_ (30:70 *v*/*v*) yielded the brown complex **2** (0.014 g, 18%) and the known green complex [(Cp*Co)(*μ*-C_3_S_5_-*κ*^1^S:*κ*^1^S′] **3** (0.012 g, 12%) [25].

**2**: MS (ESI^+^) calcd. for C_21_H_31_Co_2_S_6_^+^ [M+H]^+^ *m*/*z* 592.9414, found 592.9376; ^1^H NMR (CDCl_3_, 500 MHz, 22 °C): *δ* = 1.38 (s, 15H; 1 × Cp*), 1.63 ppm (s, 15H; 1 × Cp*); ^13^C{^1^H} NMR (CDCl_3_, 125 MHz, 22 °C): *δ* = 9.1, 10.0 (s, C_5_Me_5_), 92.4, 94.9 (s, C_5_Me_5_), 252.7 (C=S) ppm; IR (CH_2_Cl_2_): $\tilde{\nu }$ = 1020 cm^−1^ (C=S); UV–vis [CH_2_Cl_2_, *λ*, nm]: 326, 384, and 570.

### 3.2. Single Crystal X-ray Diffraction Analysis

Suitable X-ray-quality crystals of **2** and **5**/**6** were grown by slow diffusion of an *n*-hexane/CH_2_Cl_2_ solution at 5 °C. The crystal data were collected and integrated using a Bruker D8 VENTURE diffractometer (Billerica, MA, USA) with a PHOTON 100 CMOS detector (Tokyo, Japan) for **2** and a Bruker AXS Kappa APEX2 CCD diffractometer (Billerica, MA, USA) for **5** and **6** with graphite monochromated Mo-*K*α (*λ* = 0.71073 Å) radiation at 293(2) K. The structures of these complexes were solved by heavy atom methods using *SHELXS-97* [46] and *SHELXT-2014* and refined using *SHELXL-2014*, *SHELXL-2017*, and *SHELXL-2018* [47]. All the molecular structures were drawn using *Olex2* [48]. Note that complexes **5** and **6** co-crystallize in the same crystallographic unit with disorder mainly over the sulfur and carbon atoms, with 0.56 and 0.44 occupancies, respectively. The crystal structure of **6** is further disordered over two components with a site occupancy ratio of 0.31:0.13. The crystallographic data were deposited with the Cambridge Crystallographic Data Centre as supplementary publication, nos. CCDC–2111767 (**2**) and 2349360 (**5** and **6**). These data can be obtained free of charge from the Cambridge Crystallographic Data Centre via www.ccdc.cam.ac.uk/data_request/cif access on 18 April 2024.

Crystal data for **2**: C_21_H_30_Co_2_S_6_, *M_r_* = 592.67, monoclinic, space group *P*2_1_*/n*, unit cell, *a* = 10.4689(14) Å, *b* = 32.997(5) Å, *c* = 14.971(2) Å, *α* = 90°, *β* = 99.029(10)°, *γ* = 90°, *Z* = 8, *V* = 5107.6(12) Å^3^, *µ* = 14.806 mm^−1^, *F*(000) = 2448, *ρ*_cacld_ = 1.541 g/cm^3^, *R*_1_ = 0.0883, *wR*_2_ = 0.2689, 8461 independent reflections [2*θ* ≤ 64.999°] and 672 parameters.

Crystal data for **5** and **6**: 0.56 (C_22_H_30_S_6_V_2_) + 0.44 (C_22_H_34_S_6_V_2_), *M_r_* = 590.42, monoclinic, space group C2/*c*, unit cell, *a* = 18.6613(7) Å, *b* = 9.5327(4) Å, *c* = 16.0532(6) Å, *α* = 90°, *β* = 114.511(10)°, *γ* = 90°, *Z* = 4, *V* = 2598.39(18) Å^3^, *µ* = 1.211 mm^−1^, *F*(000) = 1223, *ρ*_cacld_ = 1.509 g/cm^3^, *R*_1_ = 0.0392, *wR*_2_ = 0.1066, 11180 independent reflections [2*θ* ≤ 54.218°] and 217 parameters.

### 3.3. Computational Details

Density functional theory (DFT) optimization of complex **2** was carried out using the *Gaussian 16 program* [49] with the B3LYP functional [50] and the def2-TZVP [51,52] basis set from the EMSL (Environmental Molecular Sciences Laboratory) Basis Set Exchange Library. Complex **2** was thoroughly optimized without any solvent effect in the gaseous state, starting from its X-ray crystallographic structure. Frequency calculations were carried out to check the nature of the stationary state of **2** to confirm the absence of any imaginary frequency to confirm that it was an energy minimum on the potential energy hypersurface. A natural bonding analysis (NBO) was carried out with the NBO partitioning scheme [53,54,55,56], and Wiberg bond indexes [57] were obtained from it. A QTAIM [58,59,60] analysis was performed utilizing the *Multiwfn V.3.6* package [61]. The optimized structure and orbital graphics were produced using the *GaussView* (Version 3.09) [62] and *Chemcraft* (Version 1.8) [63] software.

## 4. Conclusions

In summary, we have isolated and structurally characterized a couple of bimetallic thiolate complexes of early and late transition metals featuring both partial and full reduction of the CS_2_ moiety. One is a bimetallic cobalt thiolate complex which contains a perthiocarbonate group, as well as a disulfide group connected to two Co atoms in a *µ*_2_-*ɳ*^2^ fashion. This complex displays an interesting coordination environment around the two homometallic centers. Investigations to evaluate the possibility of other early and late transition metal thiolate complexes with rare coordination modes are underway.

## Figures and Tables

**Table 1:** Selected structural parameters and spectroscopic data of transition metal complexes featuring perthiocarbonate moiety [CE_4_]^2−^.

Bonding Modes of [CE_4_]^2−^		Structural Parameters			^13^C{^1^H} ^b^	ν_CE_ (cm^−1^)
		*d*_C–E_ (Å) ^a^	*d*_C=E_ (Å) ^a^	∠E-C–E (°)		
[*ɳ*^2^]	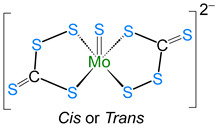	1.73 1.71	1.59 (*cis*) 1.65 (*trans*)	118.3 (*cis*) 119.6 (*trans*)	245.6 246.0	977 (*cis*) 980 (*trans*)
	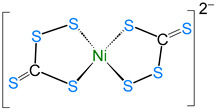	1.71	1.67	120.2	na	na
	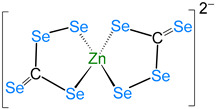	1.85	1.81	124.0	na	na
[*µ*_2_-*ɳ*^2^]	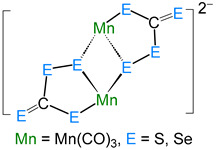	1.72 (S) 1.85 (Se)	1.67 (S) 1.81 (Se)	120.8 (S) 122.5 (Se)	na	na
	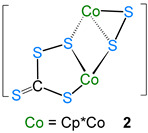	1.72	1.663(10)	119.2	252.7	1020

^a^ Average distance. ^b^ In ppm. na = Not available.

## Data Availability

The original contributions presented in the study are included in the article/supplementary material, further inquiries can be directed to the corresponding author/s.

## Supplementary Materials

The following supporting information can be downloaded at https://www.mdpi.com/article/10.3390/molecules29112688/s1. It contains ^1^H, ^13^C{^1^H}, ^11^B{^1^H}, ^31^P{^1^H} NMR, IR, and mass spectra (Figures S3–S8), X-ray analysis details, and CIF and checkCIF files, as well as the xyz coordinates of the DFT-optimized cluster **2**, calculated HOMO and LUMO energy levels and HOMO-LUMO gaps, selected bond parameters, and Wiberg bond indices (WBI) for cluster **2** (Figures S10 and S11). All pulse sequences are available in a commercial Bruker spectrometer. ^1^H decoupled ^11^B spectra were processed with backward linear prediction algorithm to remove broad ^11^B background signal from NMR probe and NMR tube. References [38,64,65,66] are cited in the supplementary materials.
