# Supplementary material for: Bimetallic Perthiocarbonate Complexes of Cobalt: Synthesis, Structure and Bonding
Source: Molecules. 2024 Jun 6;29(11):2688. doi: 10.3390/molecules29112688 (PMC11173379; doi:10.3390/molecules29112688)
Supplement: Supplementary file 1 [file molecules-29-02688-s001.zip › molecules-3018842-supplementary.pdf]

Bimetallic Perthiocarbonate Complex of Cobalt: Synthesis, Structure and Bonding

Alaka Nanda Pradhan, Shivankan Mishra, Urminder Kaur, Bikram Keshari Rout, Jean-François Halet, and Sundargopal Ghosh

SUPPORTING INFORMATION

Table of contents

II. Experimental details

Scheme S1 — Synthesis of the vanadium thiolate complexes **5** and **6**.

A. Supplementary Data

Figure S1 — Molecular structure and labeling diagrams of **5** and **6**.

Figure S2 — Molecular structure and labeling diagrams of **6**.

B. Spectroscopic Details

Figure S3 — ESI-MS spectrum of **2**.

Figure S4 — ^{1}H NMR spectrum of **2** in $CDCl_3$.

Figure S5 — ^{13}C{^{1}H} NMR spectrum of **2** in $CDCl_3$.

Figure S6 — IR spectrum of **2** in CH_2Cl_2.

Figure S7 — ^{11}B{^{1}H} spectrum of *in-situ* {**1** + CS_2 + PPh_3} reaction mixture in $CDCl_3$.

Figure S8 — ^{31}P{^{1}H} spectrum of *in-situ* {**1** + CS_2 + PPh_3} reaction mixture in $CDCl_3$.

II. Computational Data

Table S1 — Calculated Co–Co bond distances ($d_{M–M}$), M–S bond distances ($d_{M–S}$), Co–S–Co bond angles (<Co–S–Co), WBI indices of M–M bond, natural charges (q_M and q_S), natural valence population (*Pop*) and HOMO–LUMO energy gap (ΔE_{H-L}) of **2**.

Table S2 — TD-DFT calculated energies (excitation energy (eV), λ_{calc} (nm)), oscillator strength (*f*), and main composition of the first UV−vis electronic excitations for **2**. Experimental absorption wavelengths (λ_{exp}, nm) of **2** are given for comparison.

Figure S9 Absorption spectrum of **2** computed at the TD-DFT-B3LYP/Def2-TZVP level of theory (ε in $LM^{-1}cm^{-1}$).

Figure S10 Selected molecular orbitals of **2** related to most intense electronic transitions [isocontour values: ±0.045 $(e/bohr^3)^{1/2}$].

III. Cartesian Coordinates of the Optimized structure 2

Figure S11 Optimized geometry of **2**.

IV. References

I. Experimental details

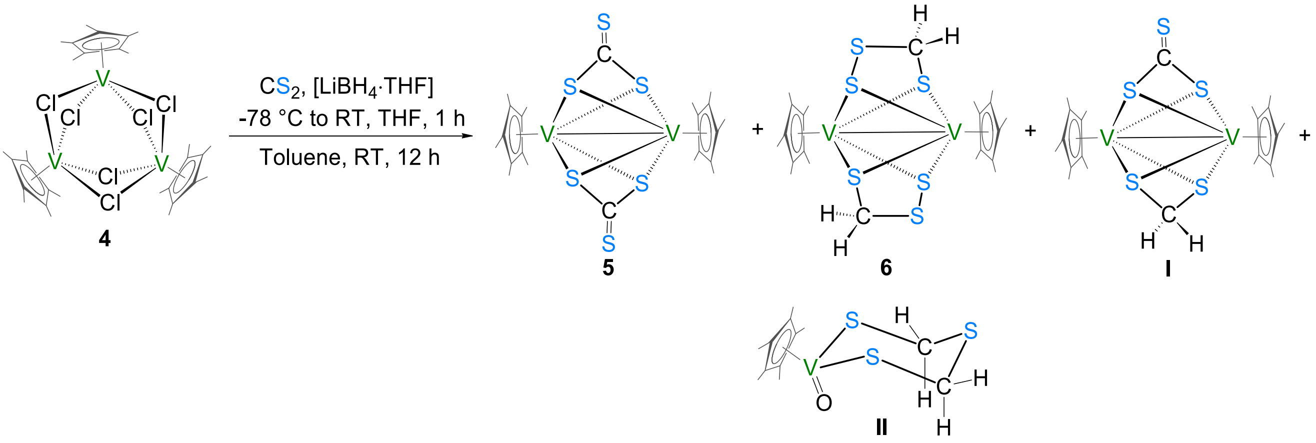

Scheme S1. Synthesis of the vanadium thiolate complexes **5** and **6**.

Synthesis of 5 and 6. In a pre-dried Schlenk tube, [Cp*VCl$_2$]$_3$ (**4**) (0.10 g, 0.13 mmol) was suspended in 10 mL toluene. To this suspension of **4**, a freshly prepared *in situ* intermediate from CS_2 and [$LiBH_4$·THF] was added dropwise at –78 °C under argon atmosphere. The reaction mixture was then kept under constant stirring at room temperature 18 hours. The solvent was removed under vacuum and the solid residue was extracted using *n*-hexane/THF (80:20 v/v) followed by separating and purifying through silica-gel coated TLC plates. Elution with *n*-hexane/toluene (80:20 v/v) yielded **5** and **6** (0.002 g, 4%) as a yellow solid along with the earlier reported yellow **I** (0.006 g, 12%) [1] and purple **II** (0.014 g, 11%) complexes [2].

A. Supplementary Data

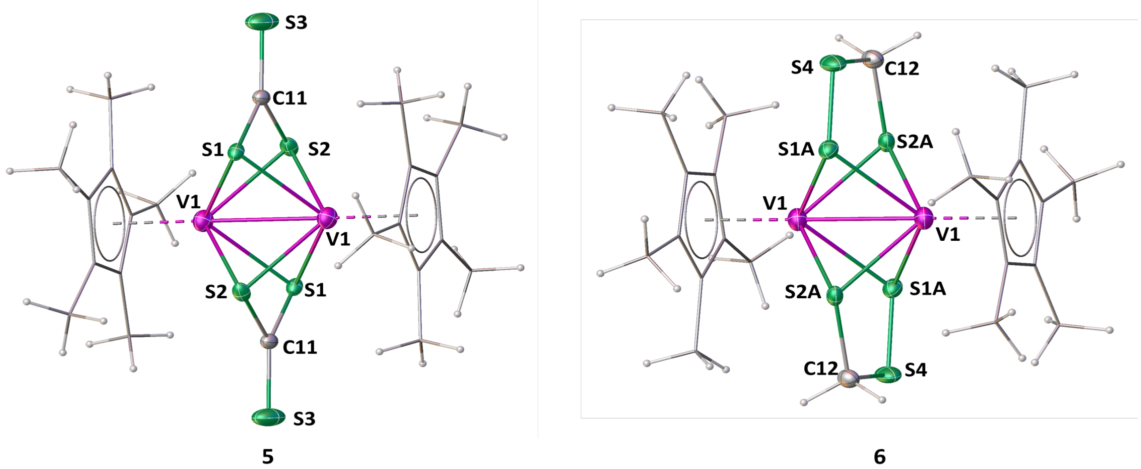

Figure S1. Molecular structure and labeling diagrams of **5** (left) and **6** (right) (co-crystallized in the same crystallographic unit). Selected bond lengths (Å) and bond angles (°): **5**: V1–V1 2.570, V1–S1 2.432(9), V1–S1 2.438(8), C11–S3 1.611(6), C11–S2 1.792(13), C11–S1 1.842(11), S1–C11–S2 105.5(5), V1–S1–V1 63.7(2), V1–S2–V1 63.3(2), S1–V1–S1 116.3(2), S2–C11–S1 105.5(5), C11–S1–V1 83.9(3), C11–S2–V1 84.4(4). **6**: V1–V1 2.570, S1A–S4 1.805(18), V1–S2A 2.43(2), C12–S4 2.045 (17), C12–S2A 1.80(3), S1A–S4 1.805(18); S2A–C12–S4 105.9(11), S1A–S4-C12 102.5(9), S4–S1A–V1 116.6(11), V1–S1A–V1 62.4(6), V1–S2A–V1 63.4(5).

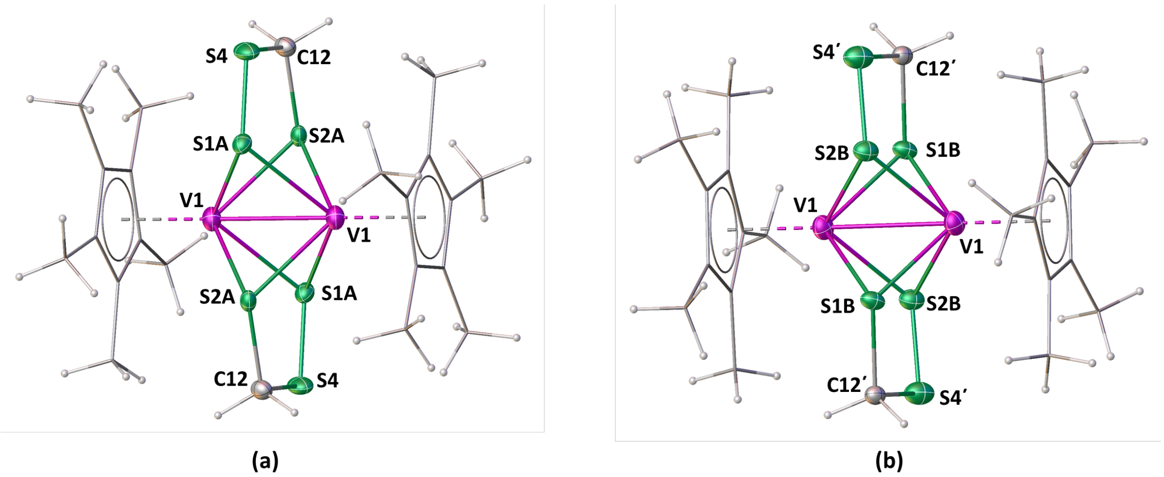

Figure S2. Molecular structure and labeling diagram of **6** with site occupancy ratio of (a):(b) = 0.31:0.13. Selected bond lengths (Å) and angles (°) are: (a): V1–S2A 2.43(2), C12–S4 2.045 (17), C12–S2A 1.80(3), S1A–S4 1.805(18); S2A–C12–S4 105.9(11), C12–S4–S1A 102.5(9); (b): V1–S2B 2.37(3), C12′–S4′ 1.93 (4), C12′–S1B 1.84(5), S2B–S4′ 1.80(2); S1B–C12′–S4′ 106.0(3), C12′–S4′–S2B 102.0(18).

I. Spectroscopic Details

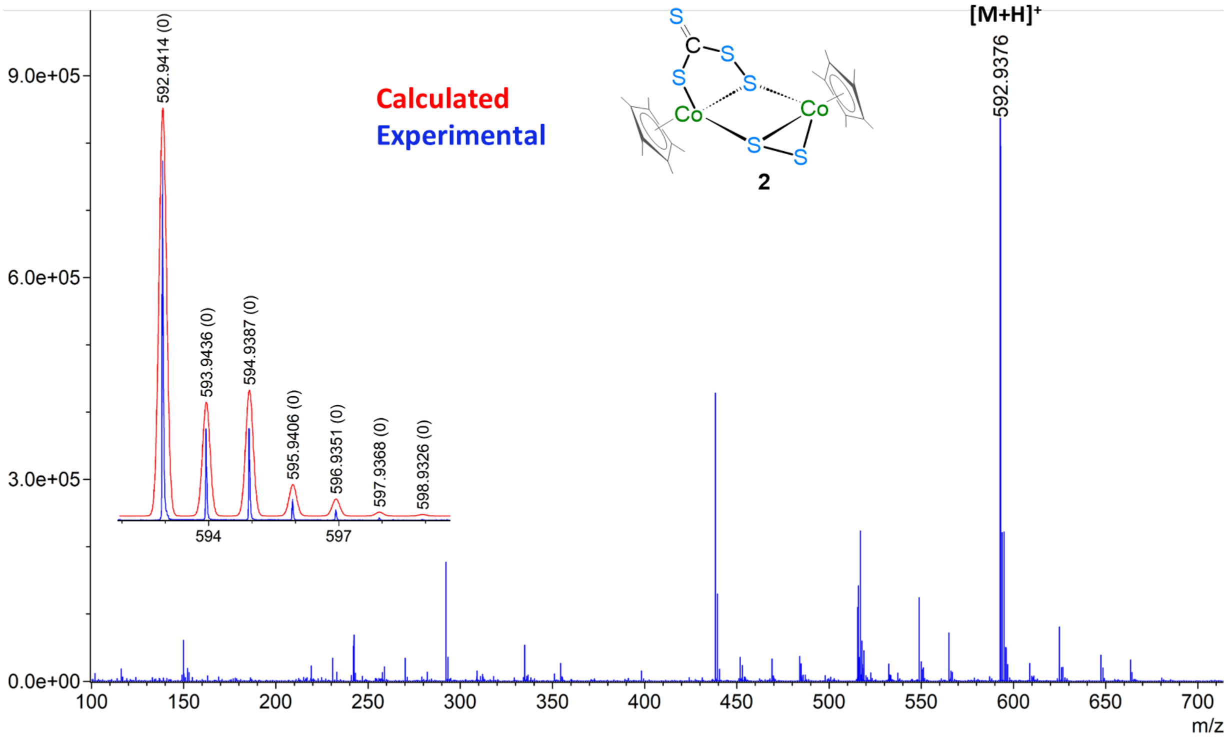

Figure S3. ESI-MS spectrum of **2**.

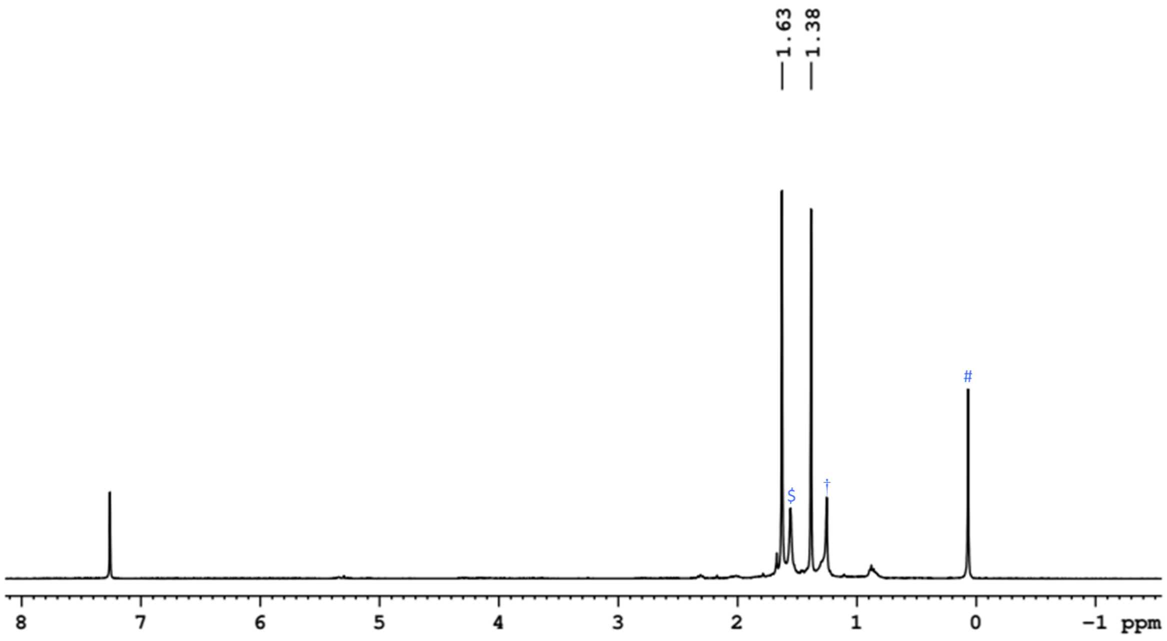

Figure S4. ^{1}H NMR spectrum of **2** in $CDCl_3$ ($$H_2O$, †hexane, #silicone grease).

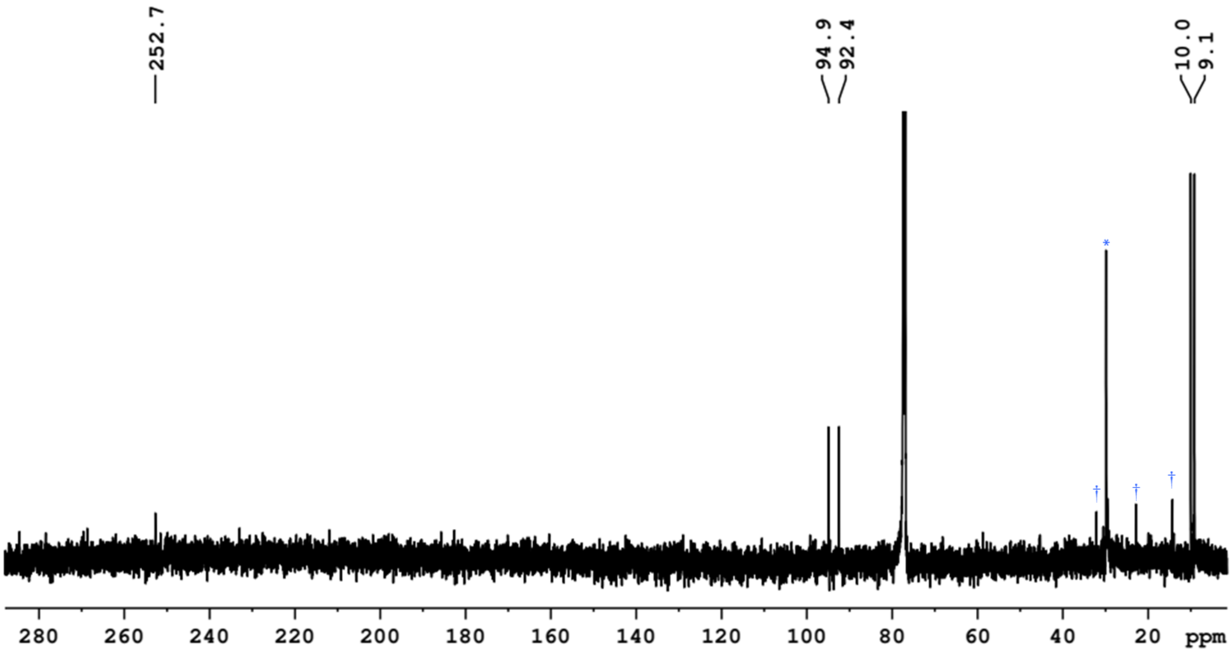

Figure S5. $^{13}C\{^{1}H\}$ NMR spectrum of **2** in $CDCl_3$ (†hexane, *H-grease).

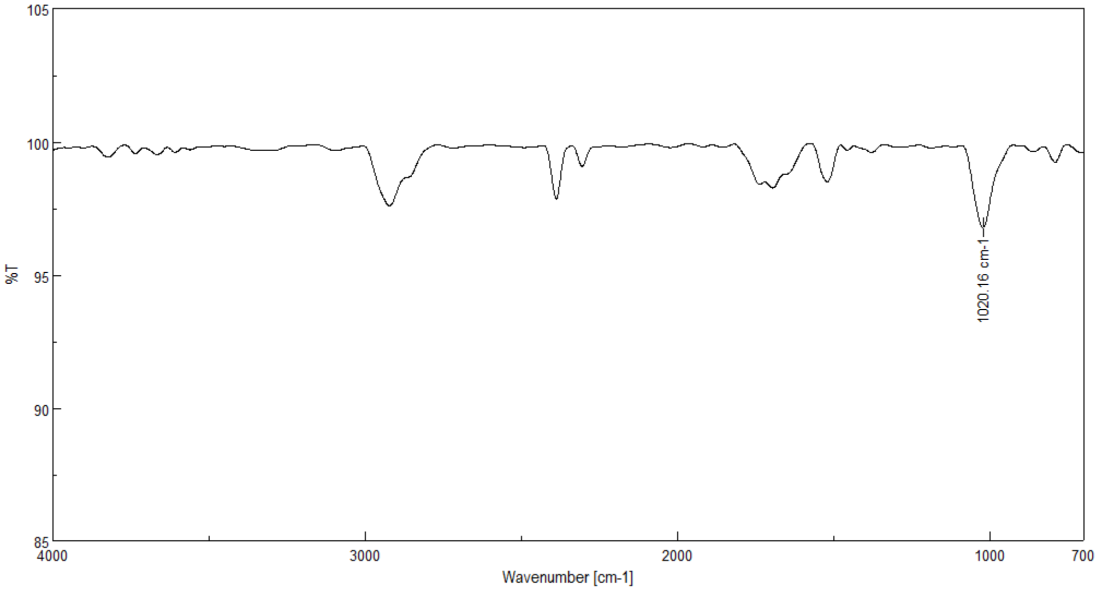

Figure S6. IR spectrum of **2** in CH_2Cl_2.

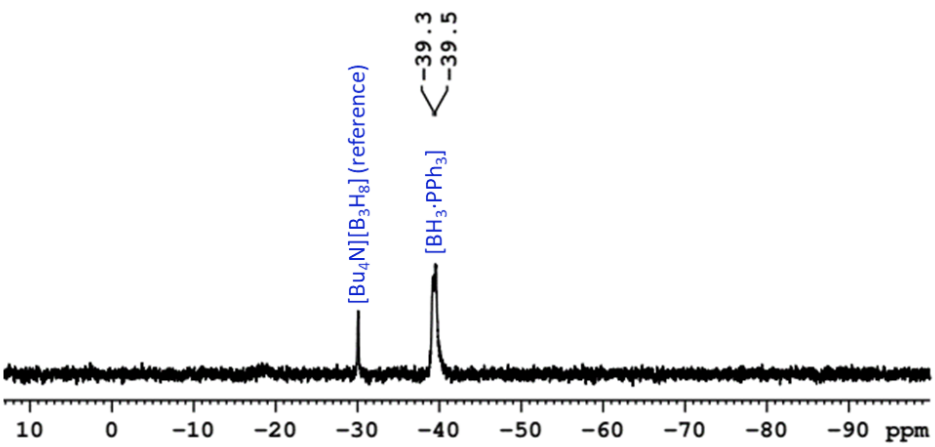

Figure S7. $^{11}B\{^1H\}$ spectrum of *in-situ* {**1** + CS_2 + PPh_3} reaction mixture in $CDCl_3$. [3-5]

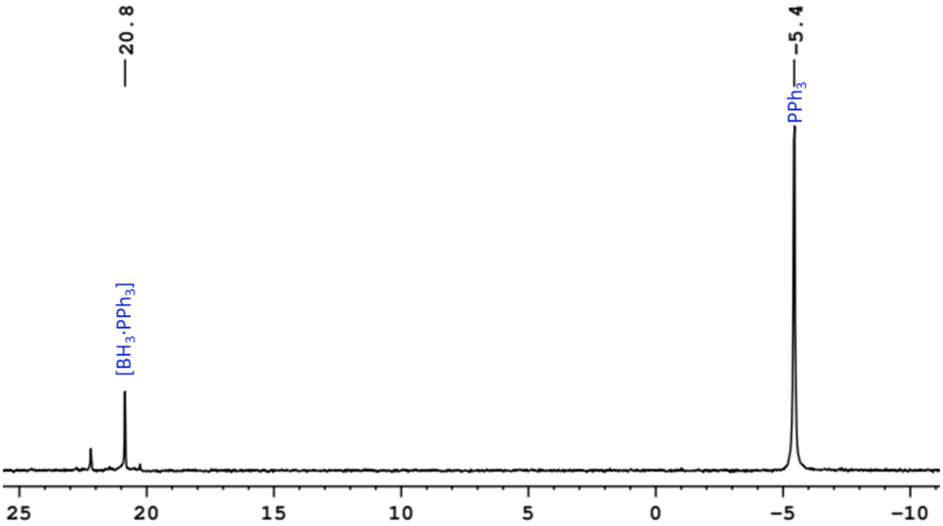

Figure S8. $^{31}P\{^1H\}$ spectrum of *in-situ* {**1** + CS_2 + PPh_3} reaction mixture in $CDCl_3$.

II. Computational Data

Table S1. Calculated Co–Co bond distances (d_{M-M}), M–S bond distances (d_{M-S}), Co–S–Co bond angles (<Co–S–Co), WBI indices of M–M bond, natural charges (q_M and q_S), natural valence population (*Pop*) and HOMO–LUMO energy gap (ΔE_{H-L}) of **2**.

d_{M-M} (Å)	d_{M-S}(Å)	<M-S-M (°)	WBI (M-M)	q_M	q_s	*Pop* (M_{val})	*Pop* (S_{val})	ΔE_{H-L} (eV)
3.390	2.267	94.832		-0.454	-0.052	9.423	6.029	3.192
	2.283	97.317		-0.369	0.192	9.335	5.754	
	2.261				0.179		5.785	
	2.320				0.146		5.807	
	2.254				0.262		5.678	
	2.264				-0.153		6.110	

Table S2. TD-DFT calculated energies (excitation energy (eV), λ_{calc} (nm)), oscillator strength (*f*), and main composition of the first UV−vis electronic excitations for **2**. Experimental absorption wavelengths (λ_{exp}, nm) of **2** are given for comparison.

No	Excitation Energy (eV)	Wavelength λ (nm)		Main electronic transition (% weight) [b]
		Calc. (*f*) [a]	Expt.	
1	2.229	556 (0.025)	570	HOMO→LUMO+3 (9)
2	2.782	446 (0.020)		HOMO-1→LUMO+1 (12)
3	2.816	440 (0.013)		HOMO-1→LUMO+3 (13)
4	2.907	427 (0.023)		HOMO-3→LUMO+1 (13)
				HOMO-1→LUMO+1 (13)
5	3.108	399 (0.023)		HOMO-2→LUMO+3 (12)
				HOMO-1→LUMO+3 (38)
6	3.126	397 (0.015)		HOMO-2→LUMO+2 (44)
7	3.248	382 (0.042)	384	HOMO-6→LUMO (11)
				HOMO-4→LUMO (12)
				HOMO-3→LUMO (15)
				HOMO-2→LUMO+4 (14)
8	3.400	365 (0.021)		HOMO-3→LUMO+1 (11)
				HOMO-3→LUMO+2 (11)

9	3.455	359 (0.019)		HOMO-5→LUMO (20)
				HOMO-4→LUMO+2 (13)
				HOMO-3→LUMO+2 (13)
10	3.474	357 (0.054)		HOMO-9→LUMO+1 (12)
11	3.564	348 (0.036)		HOMO-4→LUMO+3 (11)
				HOMO-1→LUMO+4 (14)
12	3.569	347 (0.017)		HOMO-1→LUMO+4 (52)
13	3.591	345 (0.014)		HOMO-5→LUMO+1 (7)
				HOMO-5→LUMO+2 (7)
14	3.636	341 (0.023)		HOMO-8→LUMO+2 (11)
				HOMO-3→LUMO+3 (11)
				HOMO-2→LUMO+4 (13)
15	3.697	335 (0.050)		HOMO-11→LUMO (13)
16	3.762	330 (0.025)		HOMO-6→LUMO+1 (12)
				HOMO→LUMO+5 (11)
17	3.793	327 (0.080)	326	HOMO-6→LUMO (11)
18	3.851	322 (0.027)		HOMO-7→LUMO (19)
				HOMO-4→LUMO+4 (12)
19	3.922	316 (0.038)		HOMO-7→LUMO (13)
				HOMO-6→LUMO+3 (28)
				HOMO→LUMO+5 (19)
20	3.997	310 (0.132)		HOMO-4→LUMO+4 (28)

[a]Oscillator strength greater than 0.010. [b]Components with greater than 10% contribution shown.

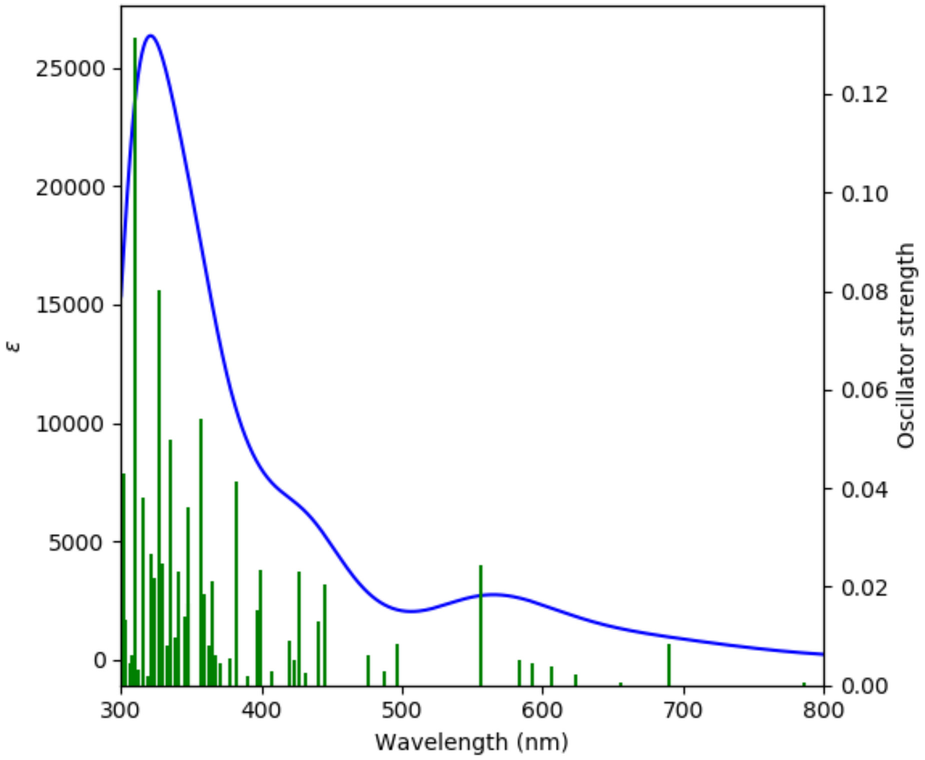

Figure S9. Absorption spectrum of **2** computed at the TD-DFT-B3LYP/Def2-TZVP level of theory (ε in $LM^{-1}cm^{-1}$).

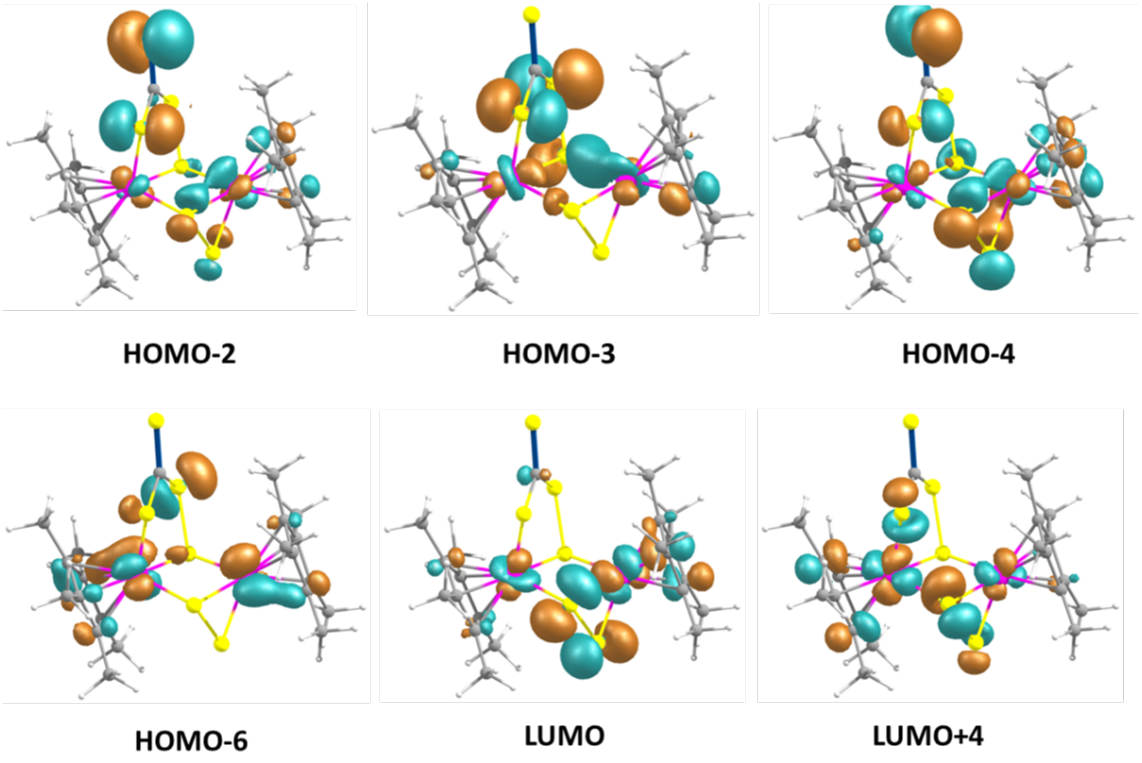

Figure S10. Selected molecular orbitals of **2** related to most intense electronic transitions [isocontour values: ±0.045 $(e/bohr^3)^{1/2}$].

III. Cartesian Coordinates of the Optimized Structure 2

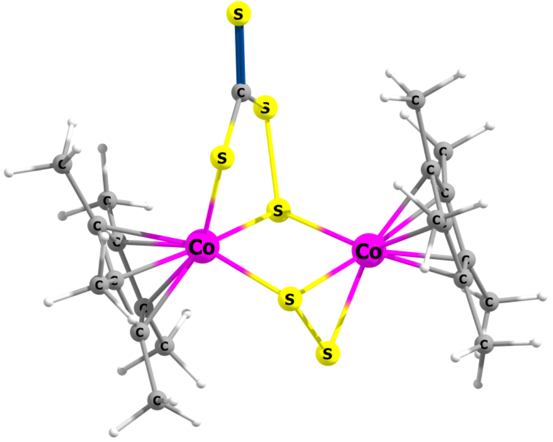

Figure S11. Optimized geometry of **2**.

Total energy = –5973.76317959 a.u.

Cartesian coordinates for the calculated structure **2** (in Å)

C	3.704523000	0.020408000	-0.047484000
C	3.255758000	-0.402533000	1.223711000
C	3.382592000	-1.011397000	-0.999181000
C	2.821446000	-2.111235000	-0.275436000
C	2.697027000	-1.726851000	1.085515000
C	2.217743000	-2.582802000	2.212717000
H	1.480667000	-3.310261000	1.878531000
H	1.755123000	-1.985537000	2.997688000
H	3.056627000	-3.126610000	2.659910000
C	3.410808000	0.333135000	2.515165000
H	4.293930000	-0.028281000	3.051929000
H	2.548955000	0.187090000	3.165675000
H	3.531203000	1.403607000	2.359525000
C	4.441642000	1.282074000	-0.354121000
H	4.191271000	1.668222000	-1.340514000
H	5.520011000	1.093133000	-0.329936000
H	4.225339000	2.066294000	0.369249000
C	3.740701000	-0.997003000	-2.451615000
H	3.670175000	0.006803000	-2.869869000
H	3.086266000	-1.645902000	-3.032902000
H	4.767773000	-1.345277000	-2.602258000
C	2.473200000	-3.447293000	-0.844934000
H	3.340345000	-4.112454000	-0.781607000
H	2.185939000	-3.380996000	-1.893459000
H	1.654745000	-3.917119000	-0.301822000
C	-3.013547000	1.257860000	-0.367469000
C	-3.370370000	0.693510000	0.914250000
C	-3.799733000	-0.643359000	0.708418000
C	-3.692381000	-0.924823000	-0.700052000
C	-3.233732000	0.258674000	-1.353192000
C	-3.038734000	0.428488000	-2.825436000
H	-2.791096000	-0.514146000	-3.311409000
H	-2.243114000	1.138880000	-3.044879000
H	-3.957819000	0.806286000	-3.285136000
C	-2.675028000	2.687650000	-0.636403000
H	-3.590057000	3.250207000	-0.851743000
H	-2.013562000	2.795473000	-1.494011000
H	-2.189476000	3.162060000	0.212719000
C	-3.377242000	1.433328000	2.212522000
H	-4.326158000	1.965278000	2.335745000
H	-2.579776000	2.173695000	2.258069000
H	-3.263331000	0.761075000	3.062049000
C	-4.334703000	-1.572917000	1.751221000
H	-3.898454000	-1.374531000	2.729432000
H	-4.136263000	-2.614350000	1.502601000
H	-5.419414000	-1.455906000	1.844586000
C	-4.104715000	-2.191051000	-1.378609000
H	-3.487824000	-2.398352000	-2.252476000
H	-5.143975000	-2.119269000	-1.715851000
H	-4.024855000	-3.048733000	-0.713915000
C	0.839071000	2.767123000	-0.224145000
S	0.925652000	4.384660000	-0.549909000
S	0.276683000	2.344258000	1.395019000
S	1.246584000	1.519937000	-1.334152000
S	-0.017712000	0.264587000	1.384970000
S	-0.172519000	-1.334569000	-1.180692000
S	-1.013811000	-2.543959000	0.254693000
Co	1.576181000	-0.368217000	-0.122772000
Co	-1.806817000	-0.430483000	0.081385000

References

1. Kaur, U.; Saha, K.; Bairagi, S.; Das, A.; Roisnel, T.; Paine, T.K.; Ghosh, S. Structural and electronic analysis of bimetallic thiolate complexes of group-5 transition metal ions. *J. Organomet. Chem.* **2021**, *949*, 121943–121954.
2. Kaur, U.; Saha, K.; Raghavendra, B.; Ghosh, S. Role of Metals and Thiolate Ligands in the Structures and Electronic Properties of Group 5 Bimetallic–Thiolate Complexes. *Inorg. Chem.* **2020**, *59*, 12494–12503.
3. All pulse sequences are available in a commercial Bruker spectrometer. ^{1}H decoupled ^{11}B spectra were processed with backward linear prediction algorithm to remove broad ^{11}B background signal from NMR probe and NMR tube.
4. Weiss, R.; Grimes, R.N. Sources of Line Width in Boron-11 Nuclear Magnetic Resonance Spectra. Scalar Relaxation and Boron-Boron Coupling in B_4H_{10} and B_5H_9. *J. Am. Chem. Soc.* **1978**, *100*, 1401–1405.
5. Led, J.J.; Gesmar, H. Application of the linear prediction method to NMR spectroscopy. *Chem. Rev.* **1991**, *91*, 1413–1426.
